# Conceptualizing community data governance for race-related, population data: a scoping review and key informant interviews

**DOI:** 10.23889/ijpds.v9i5.1666

**Published:** 2024-09-18

**Authors:** Elise Leong-Sit, Laura Ferreira-Legere, Sabella Yussuf-Homenauth, Astrid Guttmann, Baiju R Shah, Michael J Schull, Sujitha Ratnasingham, J Michael Paterson

**Affiliations:** 1 ICES; 2 Hospital for Sick Children; 3 SickKids Research Institute; 4 McMaster University; 5 Sunnybrook Research Institute; 6 Sunnybrook Health Sciences Centre; 7 University of Toronto

## Objective

There is growing recognition of the importance of community data governance to build accountability of research institutions to communities. Our organization, a steward of health and administrative population-level data, has previously implemented community governance structures for Indigenous data. This scoping initiative explores development and implementation of an additional community governance structure for race-related data.

## Approach

We conducted a scoping review of peer-reviewed and grey literature to identify existing practices of community data governance. We also conducted key informant interviews with thirteen racialized community stakeholders, who addressed open-ended questions on potential co-design processes as well as governance mandates, scopes, barriers, and facilitators.

## Results

The scoping review identified eight community data governance examples. Two of these pertained to race-related data, while the remaining six pertained to other data that identified “community” geographically, by disease condition, life stage, and/or economic circumstance. Governance structures were diverse, ranging from one-time crowd-design of a data-sharing agreement to quarterly meetings of a governing board to review project-level data requests. Key informant interviews provided four themes to guide implementation in the context of our organization: exploring organizational readiness, considering who should be involved, defining the scope and mandate, and drafting an approach and process.

## Conclusions

We are committed to implementing a community governance structure for race-related, population-level data. However, there are limited examples of similar structures in the existing literature.

## Implications

The identified examples and the advice of community stakeholders will guide co-design of a preliminary structure, scope, and mandate for community governance of race-related, population-level data.

